# The use of patient and public involvement and engagement in the design and conduct of implementation research: a scoping review

**DOI:** 10.1186/s43058-025-00725-w

**Published:** 2025-04-10

**Authors:** Amy Mathieson, Lisa Brunton, Paul M. Wilson

**Affiliations:** Centre for Primary Care and Health Services Research, The University of Manchester, Williamson Building, Oxford Road, Manchester, M13 9PL England

**Keywords:** Patient and public involvement and engagement, Implementation research, Evidence-based innovations

## Abstract

**Background:**

Most implementation research focuses on understanding and changing health professionals’ work practices. While Patient and Public Involvement and Engagement (PPIE) in applied health research is recognised as best practice, and is often a requirement for funders globally, little is known about the role of patients and the public in implementation research.

**Methods:**

Guided by Arksey and O’Malley’s framework, we conducted a scoping review to categorise PPIE in the design and conduct of implementation research, including how patients and the public have been involved, the reported impact of patient and public involvement, and the reported benefits and challenges to involving patients and the public in implementation research. We searched four databases: MEDLINE, Embase, CINAHL, and SCOPUS. To be included, studies had to report some form of PPIE in the design and conduct of implementation research. Information about each study was extracted using a structured data extraction form. Data was collated and summarised.

**Results:**

Of the 535 unique records identified, 12 were included. Analysis of the eligible studies found eight different types of PPIE activities. Researchers mostly consulted with patients and members of the public via feedback sessions, committee representation and roundtable discussions. Barriers and enablers were usually researcher related and their attempts to build, maintain, and negotiate relationships with public contributors over time. Resources and financial remuneration were also key. Most studies (*n* = 7) reported that engaging community members in the design and implementation of community-based programs and trials enhanced cultural appropriateness, and the likelihood of sustainability. However, there was little formal evaluation of the use of PPIE.

**Conclusion:**

This study can be used to design and guide future PPIE in implementation research. Given the inconsistent, and often absent, reporting of PPIE activities and barriers and enablers across the included studies, future studies should describe and evaluate the execution of PPIE in implementation research to advance practices in this field.

**Registration:**

The review was registered on Research Registry (reviewregistry1552).

**Supplementary Information:**

The online version contains supplementary material available at 10.1186/s43058-025-00725-w.

Contributions to the literature • While Patient and Public Involvement and Engagement (PPIE) has been recognised as best practice in research, very little is known about how patients and the public have been involved in the design and management of implementation research.

• This scoping review categorises PPIE in the design and conduct of implementation research promoting the adoption of evidence-based interventions into healthcare.

• The findings contribute to a recognised gap in the literature by generating a knowledge base of PPIE activities in implementation research, including the depth of involvement, benefits, challenges and impact.

## Background

Evidence-based interventions (EBIs) are often not integrated into healthcare professionals’ work, despite expectations to engage with and practice in line with evidence, and evidence based-practice being considered the foundation for the provision of quality care. Specifically, a gap remains between what is known from research and the healthcare that is provided. Reducing this “know-do gap” is a well-recognised need as delayed implementation of EBIs could negatively affect people’s health and the healthcare system [1].

One approach to bridge the “know-do gap” is to collaborate with knowledge users via involvement processes such as co-production, co-design, collaboration, involvement, engagement, patient and public involvement, community-based participatory research, and participatory action research. These terms often mean different things in different disciplines and contexts, with research priorities, roles, and outcomes differing between countries [2, 3]. For instance, in the UK, INVOLVE defines Patient and Public Involvement (PPI) as research undertaken ‘with’ or ‘by’ patients or members of the public, rather than ‘to’, ‘about’ or ‘for’ them, and ‘engagement’ is defined as dissemination of knowledge and information [4]. However, in the USA and Canada, ‘engagement’ is used in the same way as ‘involvement’ in the UK.

Most participatory research approaches engage researchers in partnership with knowledge users and focus on “knowledge for action” [5]. However, they can differ in regard to their decision-making dynamics, the intended research impact and, significantly, the partners they engage with. Both community-based participatory research (CBPR) and participatory implementation science, for example, form partnerships with all relevant parties, which can include patients, community members, community health professionals, representatives of community-based organizations, and policymakers [6, 7]. Integrated Knowledge Translation, on the other hand, is an approach to research that co-creates knowledge as the result of decision makers/funders and researcher expertise [8].

There is also international interest in involving patients as partners in research, with Patient and Public Involvement and Engagement (PPIE) in research recognised as best practice and often an essential requirement for research funding. Much attention has therefore been placed on understanding, promoting, and (to a lesser extent) evaluating PPIE in health research. As a result, the potential benefits of public involvement in research, and on researchers and patients, have been identified, such as the creation of user‐friendly information and data collection tools, appropriate and effective recruitment strategies, and enhanced dissemination of findings [9]. Recommendations are also available to help with planning, conducting, reporting and evaluating PPIE [9–12], and frameworks have been developed for this purpose [13]. While advances have been made in PPIE in health research – research largely focusing on patients – more needs to be done to understand the role of individual patients and members of the public as contributors in implementation research, which generally focuses on health professionals’ behaviour.

Implementation science is the study of methods to promote the adoption and integration of EBIs into routine health care and real world settings to improve population health, and often focuses on understanding and changing health professionals’ behaviours [14]. Whereas, implementation research is the study of the use of strategies to adopt and integrate research findings and other evidence-based knowledge into clinical and community settings [15]. The end users of implementation science and implementation research are therefore almost exclusively health professionals, with researchers and policy makers also being core audiences [16]. However, if patients are acknowledged as key partners in healthcare, they can also be targets of implementation of evidence-based practice [17]. Furthermore, most EBIs implemented within a health system have some impact on patients and the public (including potential patients and their family carers) and, crucially, patient factors can influence uptake [18–20]. It is therefore important to centre the “lived experience of patients and community members” (p. 5) receiving those interventions [16], suggesting patients and the public are relevant audiences for developing, applying, disseminating, and sustaining the evidence for implementation science and research. Moreover, patients may directly influence the behaviour of healthcare professionals in attempts to promote evidence-based practice [17].

Calls have been made to embed PPIE in the creation of best practice [21]. While a growing body of literature has combined participatory approaches and implementation science [7, 22, 23], some uncertainty still surrounds the role of PPIE in implementation research. Gray-Burrows et al. (2018) provides some guidance by asking a panel of eight PPI contributors and two researchers to independently rate PPI roles in both applied health research and implementation research. They found there was more disagreement amongst the panel regarding the role of PPI in implementation research compared to applied health research. PPI was rated as contributing less to design and management of implementation research than for applied health research. Gray-Burrows et al.’s work produced a useful framework to help guide the planning, conduct and reporting of PPI in implementation research. However, still little is known about how PPI has actually contributed to the design and management of implementation research and there is a need to categorise involvement. Furthermore, published reviews on the scope and impact of PPI on research have been limited to applied health research [9, 24–26], or PPI in the implementation of evidence-based practice for specific health conditions [27].

Thus, there is a need for a review exploring how patients and the public have been involved in implementation research, and the reported benefits to the research, researchers, and public contributors. This scoping review aims to scope the literature on the use of PPIE in the design and conduct of implementation research to categorise involvement and impact. For the purpose of this review, we define PPIE in implementation research as ‘engaging patients and the public in the co-production of knowledge to increase the likelihood that implementation efforts are useful, scalable, and sustainable in real-world settings’ [7].

## Methods

A scoping review approach was chosen as little is known about the extent to which patients and the public have been involved in implementation research. Scoping reviews are particularly useful when the existing literature is potentially diverse and emerging [28].

Arksey and O'Malley's scoping review methodology framework guided the conduct of this scoping review [29]. We followed their five-step approach: identifying the research question, identifying relevant studies, selecting studies, charting the data, and collating, summarising and reporting the results. In addition, to maximize transparency, we also followed and reported this review in line with the PRISMA extension for Scoping Reviews [30]. All procedures were pre-registered on Research Registry [31].

### Identifying the research question

The research team held several meetings to develop the research question and agree on the search terms. The following overall research question was developed: What is known from the literature about the use of PPIE in implementation research? Specifically, we aimed to map PPIE activities in implementation research promoting the adoption of evidence-based interventions into healthcare. Mapping was guided by the following questions:

1. How have patients and the public been involved in implementation research?
2. What factors have enabled the involvement of patients and the public in the implementation of evidence-based practice, and what has hindered it?
3. Does involvement vary by study design and or across geographical settings?

### Identifying relevant studies

Studies relevant to this review were identified by searching electronic databases of the published literature, including: MEDLINE (Ovid), Embase, CINAHL, and SCOPUS. A Boolean search using all the terms in Table 1 was conducted in each database. Terms were searched as both keywords in the title and/or abstract and subject headings (MeSH), as appropriate. The first author completed the final search in March 2023.

**Table 1:** Search key

1	Patient participation
2	Patient involvement
3	Patient and public engagement
4	PPI
5	PPIE
6	Co-production
7	Participatory Research
8	User-led research
9	User-centered design/user-centered design/UCD
10	Integrated knowledge translation
11	OR/1–10
12	Implement*
13	Implementation science
14	Implementation research
15	Diffusion of Innovation
16	Knowledge translation
17	OR/12–16
18	Evidence-based practice
19	Evidence-based intervention
20	OR/18–19
21	11 AND 17 AND 20

We also reviewed the reference lists of reviews and relevant primary papers to identify further records. We restricted the search to English-language papers, and did not place time restrictions.

### Study selection

The review process consisted of two levels of screening: (1) titles and abstract review, and (2) full text review. We devised an inclusion and exclusion criteria based on our research questions. For initial title and abstract screening, we focused on reports describing some form of PPIE to inform the implementation of EBIs for adult patients. If it was not possible to determine articles for full text review by screening titles and abstracts alone, we reviewed the articles’ ‘methods’ sections. We then devised further eligibility criteria for full text screening following the PCC (participant, context, concept) framework (Table 2) [32]. Studies were excluded if they only reported PPIE in the development of interventions or if they exclusively involved partners other than patients, their families, or community members, such as healthcare professionals or members of staff (e.g. lay health workers).

**Table 2:** Inclusion/exclusion criteria

	**Inclusion**	**Exclusion**
**Participants**	Studies researching the implementation of interventions related to adults; 18 years and older	Studies reporting on the implementation of interventions for paediatric populations
**Concept**	Studies reporting on PPIE activities in the design and conduct of implementation research	Studies only reporting on the implementation of interventions aimed directly at patients; Studies reporting predominately on the development of interventions, or implementation research without a detailed description of PPIE activities
**Context**	Primary and secondary care settings	
	English language	Non-English language
	Any publication type including reviews	

Systematic reviews and study protocols were eligible for inclusion. While no systematic reviews met the inclusion criteria, they were used to find potential primary studies. All records were screened in Rayyan [33]. Screening was conducted by two reviewers (AM and LB). Disagreements were resolved through discussions with a third reviewer (PW) until a consensus was reached. We also contacted the author of one paper [34] for clarification, and reviewed the protocols of two studies [35, 36] to determine eligibility.

### Data collection and charting

We extracted the following information for each study: author(s); year of publication; title; country of origin; aims/purpose; context/setting; study type and methodology; PPIE methods; who were involved; how often patients were involved; involved in the design or conduct of implementation research; innovation or intervention type; target population; use of theory; key findings (relevant to the review); barriers/challenges; facilitators to involvement; impact/outcomes/benefits; and lessons learned or things to consider. We coded the absence of information as “not mentioned”. Two reviewers (AM and LB) independently tested the data extraction table, which resulted in minor revisions. Differences in data charting were discussed and resolved through consensus.

### Data summary and synthesis

Synthesis involved descriptive analysis of study characteristics and qualitative analysis of PPIE activities, barriers and facilitators, reported impact, and lessons learned. We used an inductive approach to code the data. Two reviewers (AM and LB) reviewed the extracted data and generated themes and sub-themes. Data were then coded in Nvivo 12 plus. Both reviewers met frequently to discuss data analysis and synthesis.

## Results

**Fig. 1:**
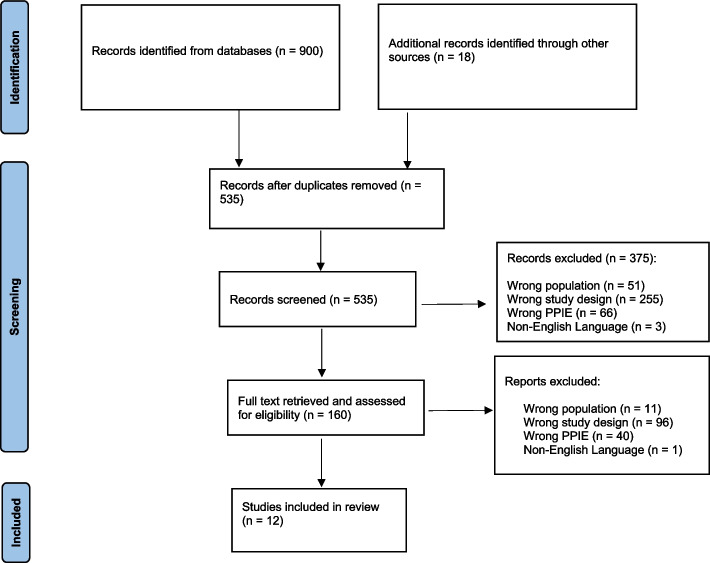
PRISMA flow diagram

The search yielded a total of 918 articles. After excluding duplicates, 535 titles and abstracts were reviewed for inclusion. Of these, 160 articles progressed to full-text review and 12 met the inclusion criteria (Fig. 1).

### Study setting

Study characteristics and descriptive findings are summarised in Table 3. Eight of the 12 studies were conducted in North America: six in the USA and two in Canada; two studies were conducted in the UK; one study was conducted in Australia; and one in Nigeria. Across all countries, EBIs were mostly implemented in community settings (*n* = 8), including nursing homes, homesteads, churches and universities. Two studies were conducted in hospital settings, and two studies were conducted in mental health settings.

**Table 3:** Study characteristics and descriptive findings

Study	Description of paper	Partners involved	PPIE activities	Depth of involvement (Design, conduct or both)	Methods	Innovation/intervention	Country	Setting
**Becker et al. (2017) **[45]	Paper describes the diverse stakeholder partnerships that have advanced the research and dissemination/implementation of The Body Project worldwide to help other intervention developers establish effective partnerships	Universities, sorority members, and eating disorders not-for-profit organisation	Provide feedback & user testing	Design	Community participatory research	The Body Project, evidence-based body image prevention program for young women	USA	Community
Delafield et al. [36]	Paper describes a community-to-community mentoring model that extends the application of community-based participatory research values, principles, and practices used in intervention development to intervention dissemination	Members from 5 partnering communities and one Community-based Organisation of community members	Community Advisory Board & mentoring partnership	Both	Community-based participatory research and the Community-to-Community Mentoring (CCM) model	The Partnerships to Improve Lifestyle Interventions (PILI), an 11-year community based participatory research initiative to eliminate obesity disparities in Native Hawaiians and Pacific Peoples	USA	Community
**Haines et al. (2022) **[37]	Paper details authors’ use of ethnography and user-centred design to describe context and apply the data towards facilitating implementation	5 young adult representatives; academic researchers in cancer care delivery and user centred design; and providers in local implementation context (NCCH) (n = 8)	Providing feedback; Roundtable discussions; and members of design team	Design	Ethnography and User-Centered Design	Young Adult Needs Assessment and Service Bridge (NA-SB), a patient reported outcomes based clinical intervention to assess and address unmet needs of young adults with cancer	USA	Hospital
**Henderson et al. (2014) **[40]	Paper describes the strategies, barriers and facilitators used in the Connections research program and identifies implications for future integrated knowledge translation efforts	Executive directors, program managers, and front line staff from addiction agencies serving women and individuals with lived experience, administrators, policy makers, and representatives from other relevant local, provincial, and national organizations	National Advisory Committee & provide feedback	Design	Integrated knowledge translation	"Connections"research program, develops and evaluates knowledge translation and exchange strategies to facilitate evidence-informed decision making in women’s services for substance abuse issues	Canada	Mental Health
Huang et al. [41]	Paper describes the context and design of the PATIENTS program. The authors reflect on experiences of implementation and present preliminary evidence of success. Describes lessons learnt in developing innovative research partnerships, expanding patient-centred outcomes research capacity, and disseminating findings for potential application to future similar studies	Three patient advisors, and representatives of partner organizations (faith-based organizations, healthcare systems, and research practitioners)	Provide feedback & External advisors to the internal steering committee	Both	Patient-centered outcomes research	PATient-centered Involvement in Evaluating the effectiveNess of TreatmentS (PATIENTS), an innovative capacity building project, which focuses on improving patient-centered outcomes research methods and addressing health disparities. Program components address training needs, bi-directional engagement, cultural competency, and dissemination and implementation. Activities include providing resources, conducting patient-centred outcomes research projects, engaging community members and disseminating findings	USA	Hospital
**LaMonica et al. (2019) **[34]	Paper describes an implementation science protocol to systematically guide implementation of technology enabled solutions in mental health services	Service users, families and carers, health professionals, and administrators	User testing; Provide feedback; and Service mapping	Design	Participatory design, knowledge translation, user testing, and rapid prototyping methodologies	InnoWell Platform, a customizable digital tool to assist assessment, monitoring and management of mental health issues and maintenance of well-being	Australia	Mental Health
**Manley et al. (2023) **[44]	Paper reports the outcomes of a participatory workshop that sought to better understand the challenges, barriers and opportunities that currently exist within the care pathway for survivors of traumatic brain injury	Three charities representing people with TBI, carers, one family member, clinicians, service users, researchers and commissioners	Roundtable discussion (workshop)	Design	Co-production and practice development methodology	Care pathway for survivors of traumatic brain injury	UK	Community
**Nápoles et al. (2012)** [42]	Paper describes the community-based participatory research methods used to develop and implement the Nuevo Amanecer program and the unique considerations in implementing a randomized controlled trial to test the program in community settings	Latina cancer survivors, advocates, oncologists, and social service providers, including representatives from the CBO partners	Community Advisory Board; Provide feedback; and Co-PI with lived experiences	Both	Community-based participatory research method	The Nuevo Amanecer program, a culturally-tailored, peer-delivered cognitive behavioral stress management intervention	USA	Community
**Odukoya et al. (2020) **[38]	Paper describes the protocol for the development of the cultural adaptation and pilot testing of two combined evidence-based interventions in church-based settings in Lagos, Nigeria. It also describes the development of an additional component (faith-based text messages) into one of the treatment arms	Representatives from the diocesan leadership and diocesan medical society, church members, and academic research team	Provide feedback; Steering committee; Focus groups with steering committee members; and ‘In house implementation team’	Design	A three-arm cluster-randomized pilot trial	Body and Soul, and the Healthy Body Healthy Spirit, 2 evidence-based interventions in church settings including individual education, group education, campaigns and promotions, church events and supportive relationships	Nigeria	Community
Sampson et al. [39]	Paper describes the protocol for the pilot cluster randomized controlled trial which includes assessing the effectiveness of the implementation strategy and exploring whether the intervention would be sustainable outside of the trial context	Two carer reference panels, each comprises up to eight family carers of people with dementia and a person living with dementia. The carers are supported by the Alzheimer’s Society research volunteer network	Carer Reference Panel	Both	Cluster randomised pilot trial with process evaluation	Interventions to Reduce Acute Care Transfers (INTERACT), a complex intervention (3 components: early warning tool; care pathway; structured communication with primary care) that aims to detect and diagnose a range of medical conditions in residents recently discharged from hospital to skilled nursing facilities and reduce readmissions	UK	Community
**Sprague-Martinez et al. (2020) **[43]	Paper describes the community engagement approach and the data driven coalition planning process incorporated in the Communities That Heal intervention, and shares early lessons and challenges related to implementing the intervention	Staff from state agencies, people with lived experience with opioid use disorder, and frontline workers	Community Advisory Board; Community engagement; and Coalition building	Both	Community-based participatory research method	Communities that HEAL (CTH) intervention, a community-engaged, data-driven planning process. Includes 3 components: community engagement, a menu of evidence-based practices to address opioid use disorder and deaths, a set of communication campaigns to reduce stigma and drive demand for evidence-based practices	USA	Community
**Wurz et al. (2021)** [35]	Paper describes 3 case studies of physical activity implementation among diverse populations to compare cases, identify common factors that affect implementation, provide practical tips and define recommendations to encourage research efforts to close the knowledge-to-practice gap	Cancer survivors, healthcare providers, and support persons	Provide feedback & semi-structured interviews with stakeholders	Both	Not mentioned	Clinic-to-community physical activity program, 10–12-week physical activity program 2-times per week for 60 min per session, focussed on full body strength, aerobic exercises, flexibility training and behavior change coaching	Canada	Community

### Time period

Among the 12 articles, eight studies were published between 2016 and 2021. Two studies were published before 2016: in 2012 and 2014. Two studies were published after 2021: one in 2022 and one in 2023.

### Methods

Most studies involved and engaged patients and members of the public through co-design and participatory methods (*n* = 7), including formal CBPR methods and Integrated Knowledge Translation. Two studies were cluster randomised pilot trials, with one utilising a hybrid trial design. One study used ethnography and user-centered design (UCD), and one study was patient-centred outcome research (PCOR).

Half of the included studies (*n* = 6) reported using a defined framework or theory to inform research design and/or implementation [34, 36–39]. Theories included, but were not limited to, the PARiHS Framework, Diffusion of Innovation Theory, Quality Implementation Framework, and the RE-AIM Framework.

### Partners involved

All studies involved multiple partners, including healthcare professionals. However, when lay people were involved, researchers most frequently engaged with users or patients with lived experience [34, 35, 37, 40–43]. Four studies engaged with family carers or family members [34, 35, 39, 44], and three studies involved community members [36, 38, 45].

### PPIE activities

**Fig. 2:**
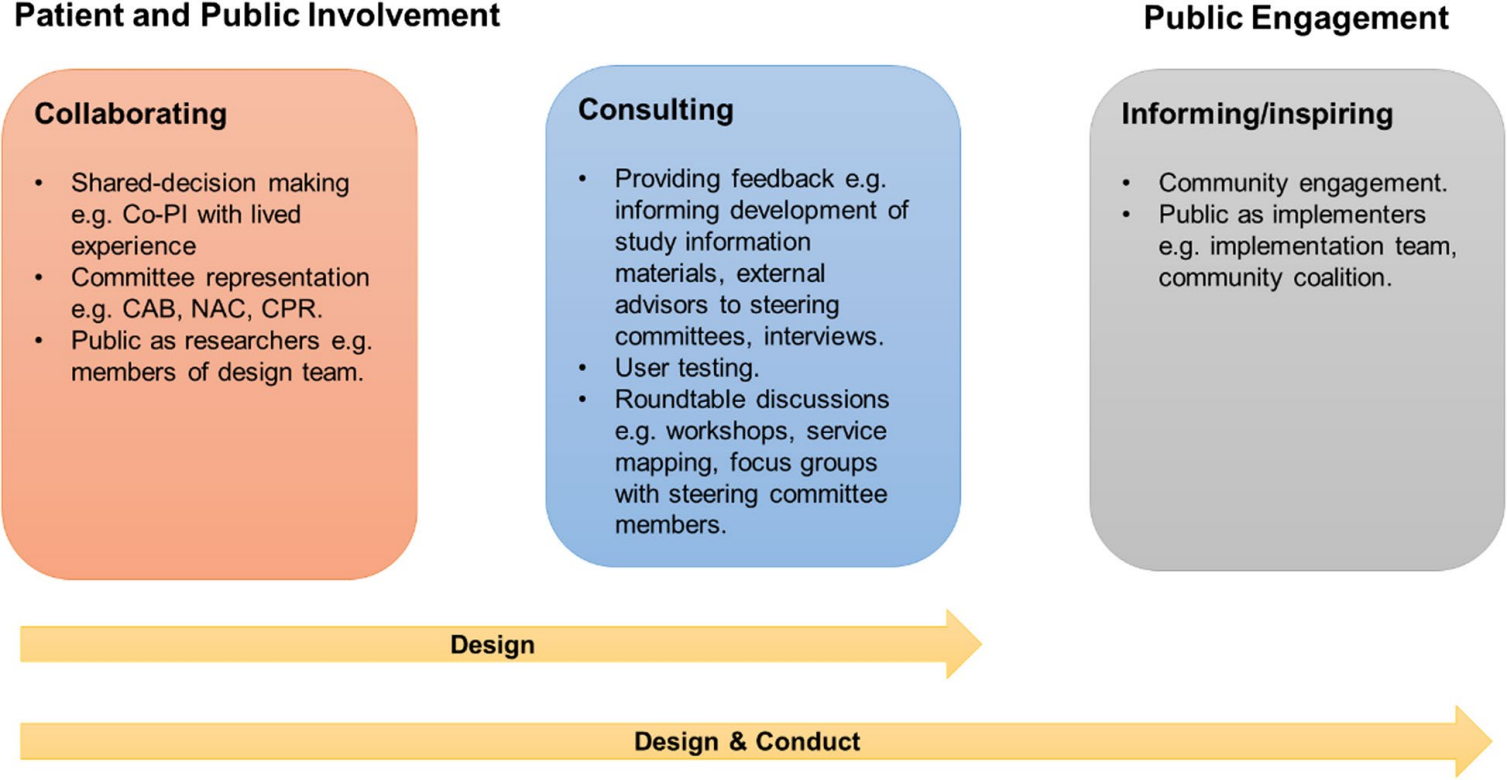
PPIE activities in implementation research

Analysis of the 12 eligible studies identified eight different types of PPIE activities, which we have further categorised into three groups: ‘collaborating’ (co-producing and shared decision making with collaborators), ‘consulting’ (collaborators informing decision making), and ‘informing/inspiring’ (stimulating thinking among other relevant parties) [46] (Fig. 2). Several studies used multiple activities.

Committee representation was a common *collaborating* approach for involving patients and the public, used by five studies. These studies mostly had a Community Advisory Board (CAB) [36, 42, 43] or a National Advisory Committee (NAC) [40], which included membership from participating communities and/or people with lived experience, to guide implementation research and outreach. Sampson et al. created two Carer Reference Panels (CRPs) to ensure public involvement at all stages of the implementation research. The CRPs also worked collaboratively on recruitment and consent processes, accessibility of information leaflets, data collection, interpretation and dissemination [39].

Two studies further *collaborated* with people with lived experience. A Co-Principal Investigator (Co-PI) on Nápoles et al. study was a clinical psychologist and breast cancer survivor. This study also had a CAB, which comprised Latina cancer survivors, advocates, oncologists, social service providers, and representatives from the Community-based Organisation (CBO) partners. The Co-PI was also the Executive Director of one of these CBOs. Haines et al., on the other hand, included patient representatives on their “design team”. Patient representatives participated in the "design team workshop" to discuss implementation strategies and potential barriers. With the design team's input, researchers also developed a set of questions for the guided walks and semi-structured interviews.

Overall, projects mostly *consulted* with patients and members of the public. Specifically, incorporating opportunities for patients and the public to provide feedback was the most frequently used approach across the studies (*n* = 7). This included establishing partnerships with community representatives to pilot the innovation and holding feedback sessions to inform implementation [45]; prototyping workshops with users [37], and providing opportunities for regular user-testing feedback from individuals with lived experience to inform on-going implementation [34]; informal input-seeking from patient representatives via individual consultations or written feedback [40]; formal input-seeking from patient and carer representatives via semi-structured interviews [35], or focus groups with steering committee members [38]; and patients as external advisors to the internal steering committee [41].

In addition to providing feedback and committee representation, roundtable discussions were often used to *consult* with patients and the public. Different roundtable discussion activities were used across four studies. Manley et al. conducted a three hour co-production workshop with twenty‐five individuals, including one family carer, to explore ways of creating and implementing a more effective care pathway for people who have experienced a traumatic brain injury. Similarly, LaMonica et al. conducted service mapping workshops with individuals with lived experience. In the service mapping workshops, participants worked collaboratively with the researchers to map the current service user journey and highlight gaps or limitations of the current care pathway, to inform design and implementation of the innovation ‘InnoWell Platform’. Two other studies also conducted roundtable discussions [37, 38]. As discussed above, Haines et al. held design workshops, and Odukoya et al. conducted focus groups with steering committee members.

Three studies used members of the public as implementers to *inform* and *inspire* others to implement the respective innovations. Delafield et al. used a Community-to-Community Mentoring model, in which community partners supported other CBOs in their adoption, implementation, and maintenance of the ‘Partnerships to Improve Lifestyle Intervention’. One of these CBOs was a group of community members who came together to offer a lifestyle intervention for fellow community members. Similarly, Odukoya et al. had an ‘in house implementation team’, which included community members, and Sprague-Martinez et al. formed community coalitions, whereby partners, including individuals with lived experience, examined local data and community assets to select and support the implementation of evidence-based practices.

### Depth of involvement and engagement

Six studies involved patients and the public in the design of implementation research, and six studies incorporated PPIE in both the design and conduct of implementation research. No studies involved patients and the public in the conduct of implementation research only. Notably, most of the studies that utilised PPIE in the design of implementation research, *consulted* with patients and the public, meaning researchers obtained feedback via roundtable discussions, user-testing workshops, written feedback requests, and interviews. Whereas, the studies that had PPIE in the design and conduct of implementation research mostly *collaborated* with patients and the public via committee representation. Two of these studies also had public members as implementers. Overall, studies utilising co-design and participatory methods [34, 36, 40, 42–45] were more likely to *collaborate* and *consult* with patients and the public. These studies were also more likely to use community members as implementers to *inform* and *inspire* others to implement interventions.

Levels of involvement differed between, and within, studies. Two studies consulted patients and the public on one occasion [40, 44], and one study held two feedback sessions [45]. The committees in two studies met yearly [40, 42], and the CAB in another study met at six-monthly intervals [39]. In two studies, patients and members of the public had quarterly meetings with researchers [34, 41]. One study reported that the CAB met monthly [36], and another study planned to hold “regular meetings” with collaborators [38]. Three studies did not provide any details regarding the frequency of involvement [35, 37, 43]. The authors of one study, which solicited on-going feedback, reported involvement was often sporadic [40].

### Barriers and enablers

Reporting of barriers and enablers varied between studies (Table 4). Only four studies outlined barriers and challenges to executing their PPIE in implementation research [41–43, 45]. Across these studies, we identified five barriers: limited time to build relationships; maintaining trust and engagement over time; managing power differentials; balancing competing interests between researchers and community members; and resource constraints.

**Table 4:** Barriers and enablers

		**Studies (N)**	**References**
**Barriers**	Limited time to build relationships	3	41, 42, 45
	Maintaining trust and engagement over time	2	43, 45
	Managing power differentials	2	41, 43
	Balancing competing interests between researchers and community members	1	41
	Resource constraints	3	40, 41, 45
**Enablers**	Funding support and financial remuneration	5	37, 38, 40–42
	Regular contact, rapport and trust between researchers and partners	6	36–38, 40, 41
	Preparation to empower partners	2	37, 44

Building relationships with partners was widely considered important to ensure evidence-based practices align with community priorities. However, three studies discussed how time consuming it was to develop these partnerships [41, 43, 45]. Sprague-Martinez and colleague’s study highlighted the challenge of cultivating relationships if the study has an ambitious timeline. Time constraints could also pose a challenge for research staff who were trying to navigate power dynamics [41, 43]. Huang et al. outlined the challenge of addressing perceived power differentials while balancing the interests of both the community and the research team. Sprague-Martinez et al. also described how the impact of the COVID- 19 pandemic and the shift to online meetings may have exacerbated pre-existing power imbalances as insufficient internet speeds in some communities led to limited engagement. Maintaining partnerships and trust overtime is therefore challenging.

Three studies highlight a lack of funding, resources, or capacity as a barrier to conducting PPIE in implementation research [40, 41, 45]. As discussed, developing partnerships is time-consuming; however, researchers’ time is often not compensated during the early stages of partnership development [45]. Patient and public involvement may also be affected by a lack of time, and CBO participation may be impeded by resource constraints, staff turnover, and agency and funder priorities [40].

We identified three enablers of PPIE in implementation research: funding support and financial remuneration; regular contact, rapport and trust between researchers and partners; and preparation to empower partners.

Five studies reported using financial remuneration to facilitate involvement. Across these studies, four types of financial remuneration were reported: payments to individuals to attend workshops or meetings [37, 41]; payments to organisations involved in the studies [38, 42]; reimbursement for travel costs [40]; and providing ‘back fill’ funding to enable staff to attend events [40]. Notably, two of these types of financial remunerations were not directly given to public contributors, rather participating organisations and staff members, which indirectly facilitated lay persons’ involvement. For example, Odukoya et al. paid churches $100 to retain committee members, which included lay people.

Building relationship with partners at the beginning of the study and regular contact throughout the research process was also considered a facilitator for PPIE in implementation research. Communication ranged from one-to-one interactions with potential PPIE members to group meetings to build rapport among public contributors [37], and engagement events encouraging capacity building and networking [38, 40]. Two papers [35, 41] also described using newsletters, briefs, webinars, meetings, and maintaining a social media presence to ensure ongoing and open communication with partners. Furthermore, Delafield et al. described the importance of bi-directional lines of communication in the form of ongoing conversations to facilitate trust, co-learning, and community relevant innovation adaptations.

Two studies that held workshops with patients and members of the public described preparatory work to facilitate involvement and empower contributors [37, 44]. Both Haines et al. and Manley et al. provided workshop attendees with material in advance to prepare for the workshop, and to enable the contributors to make an informed decision about whether to participate. Contributors were also told what to expect in the workshop, and what preparation was required. Furthermore, Manley et al. described processes to facilitate involvement in the workshop. These included ensuring contributors’ anonymity was maintained in relation to outputs from the workshop. Manley et al. also highlighted the importance of creating a safe environment to enable everyone’s voice to be heard. This involved agreeing ground rules within the group to support “openness, honesty, creativity and learning for shared mutual understanding” (p.873).

### Impact of involvement and engagement

After analysing the eligible studies, we conceptualised three categories of impact: impact on research; impact on the implementation of EBIs; and (potential) impact on community members (Table 5).

**Table 5:** Impact of involvement and engagement

Impact category	Impact	Study (N)	References
**Impact on research**	Informed (and improved design of) future research	5	36, 40–42, 44
	Improved recruitment and participants’ experiences	1	42
**Impact on implementation of EBIs**	Changed delivery model	3	37, 40, 45
	Culturally tailoring EBIs so innovation is more compatible and sustainable	7	34–36, 41–44
**Impact on community members**	Capacity Building	1	36
	Positive and empowering experience	1	44

### Impact on research

Across the included studies, the use of PPIE reportedly informed, and improved the design of, future research [37, 40–42, 44]. Henderson et al. reported that relevant research questions raised by members of the advisory committee, and for which sufficient evidence did not exist, became the basis of future research funding proposals. Similarly, Huang et al. found their PATIENTS program, which encouraged academic partners to co-develop proposals with community partners, has increased the number of PCOR proposals, including community members submitting proposals themselves. The PATIENTS program also expanded PCOR capacity by identifying the knowledge and skills gaps of academic researchers and community members. Moreover, Haines et al., Manley et al., and Napoles et al. all reported that their experiences collaborating with community members could equip implementation practitioners with the information they need to promote implementation in future studies. Specifically, Haines et al. argue that embedding ethnographic methods within UCD could help implementation science prioritise implementation determinants. Whereas, the co-produced framework model that emerged from Manley’s et al. workshop could offer a template for other neurological rehabilitation services that are likewise in need of reform.

The use of PPIE in Nápoles’ et al. study was also critical to overcoming recruitment and program delivery challenges. Nápoles et al. argued the academic-community partnership facilitated the negotiation of important trade-offs between internal and external validity, while maximizing program adoption. For example, the study team had difficulty accepting randomisation when they saw women’s distress. However, through discussions with the CAB, consensus was reached that a wait-list control group was an acceptable compromise as it allowed for stronger evidence of the program’s effectiveness and provided full access to the intervention at a later date [42].

### Impact on implementation of EBIs

Seven studies reported that patient and public involvement ensures EBIs are culturally tailored to the context in which they will be used; resulting in innovations that are more compatible and therefore sustainable [35, 36, 41–45]. Specifically, by seeking the opinions and expertise of partners, researchers were able to tailor intervention messaging to fit with partners’ values and address perceived implementation barriers [37, 45]. For example, the design team in Haines’ et al. study discussed future implementation of the intervention, anticipating barriers to implementation and brainstorming strategies to address barriers. Yet, Manley’s et al. workshop enabled participants to identify and share core priorities and enabled development of an implementation and impact framework to guide integrated services for people with traumatic brain injury at both the micro and macro level. Four of the seven studies stated that engaging community members in the design and implementation of community-based programs improves the likelihood of sustainability and the uptake of the programs by other CBOs [35, 36, 42, 43]. Furthermore, two studies reported that the consideration of cultural and contextual factors was seen by potential adopters as a relative advantage, and was important to partners [36, 41].

Three studies also reported changing the delivery model of the intervention based on feedback from public contributors [37, 40, 45], which encouraged adoption. As a result of using community participatory research methods, and after feedback from sorority members, Becker et al. switched to a train-the-trainer model for implementation of ‘The Body Project’ innovation, which decreased costs and increased sustainability. Similarly, in response to collaborators’ feedback, Henderson et al. altered the planned intervention to include a 12-month trial of a Connections’ Knowledge Broker to provide more ‘on-the-ground’ support. As discussed, Haines et al. argued that embedding ethnographic methods within UCD could help tailor interventions and implementation strategies. Specifically, Haines et al. engaged users in the analysis of contextual needs, which was considered advantageous as they did not rely solely on researchers’ interpretation.

### Impact on community members

Two studies reported potential positive impacts on community members. Delafield et al. described how fostering partnerships impacted community capacity by expanding resources to address shared social problems. Manley’s et al. evaluation also demonstrated that the workshop was a positive, collaborative and empowering experience for participants.

## Discussion

This review contributes to the existing knowledge of PPIE by exploring the range of PPIE activities in implementation research promoting the adoption of EBIs into healthcare. While participatory research approaches such as CBPR (an umbrella term for participatory approaches committed to work in partnership with members of marginalized communities) share some commonalities with PPIE, our focus on studies with implementation outcomes sheds new light on patient and public partnerships in research (Table 6). Furthermore, participatory research is a research approach that can be applied to a range of interventions drawing on various research paradigms, methodologies, and methods [6]. The boundaries between approaches is therefore not always clear, and distinctions between research paradigms are not universally accepted. This makes evaluating approaches challenging [6]. However, our scoping review, by focusing on studies which have engaged patients and the public in the co-production of knowledge to increase the effectiveness of implementation efforts, has generated a knowledge base of PPIE activities in implementation research. This includes the depth of involvement and engagement, impact of this PPIE, barriers and enablers, and the goals or purpose of these approaches.

**Table 6:** Comparing participatory approaches with review findings

	**PPIE in health research**	**CBPR**	**PPIE in implementation research**
Aim	To meaningfully involve and engage patients and the public at all stages of research	To co-create knowledge that is the result of knowledge user and researcher expertise	To co-produce knowledge to increase the likelihood that implementation efforts are useful, scalable, and sustainable in real-world settings
Intended impact	Enhance the relevance and quality of the research	Social change	Enhance the relevance and quality of the research, and make sure EBIs are culturally tailored to the context to ensure sustainability
Partners	Patients and members of the public (excluding healthcare professionals)	All relevant parties (including healthcare professionals)	Multiple partners including patients with lived experiences, family carers/family members, and community members
Level of involvement	Partners involved in any/every stage of the research process	Partners involved in any/every stage of the research process	Partners involved in any/every stage of the research process
Ethics	Research undertaken ‘with’ or ‘by’ patients or members of the public with lived experiences of the problem being addressed, rather than ‘to’, ‘about’ or ‘for’ them	Underpinned by a commitment to work in partnership with members of marginalized communities to reduce/eliminate injustices and/or inequities	Addressing problems meaningful to the user, positively impact community capacity, and empower participants

We identified eight PPIE activities, which we grouped into three categories (Fig. 2). These could be used to design and guide future PPIE in implementation research. Previous attempts have been made to identify operational definitions of public involvement in health and social sciences research. A key study is that of Hughes and Duffy (2018), in which they conducted a concept analysis exploring and clarifying the nature and meaning of public involvement in health and social sciences research. Five definitions were developed from their analysis: undefined involvement; targeted consultation; embedded consultation; co-production; and user-led research [47]. According to these definitions, most of the studies in our review used “targeted consultation” and “embedded consultation”, whereby service users from community organisations with academic partnerships were consulted on one or more occasion on aspects of the implementation research or throughout the research cycle. Only one study used “collaboration and co-production”, and no studies were “user-led research”. While most of the papers in Hughes and Duffy’s review fell under the definition “undefined involvement”, our findings suggests that PPIE in implementation research is not as defined as PPIE undertaken in other areas of applied health research. We would argue future implementation researchers could more explicitly involve and engage patients and the public throughout the research process. Specifically, patients and the public could be more involved in the *conduct* of implementation research.

Despite the need for implementation researchers to more explicitly incorporate, and evaluate, PPIE in their research, there are challenges. We found time constraints posed a challenge for researchers when developing partnerships, particularly when navigating power differentials and balancing competing interests between researchers and community members. Arguably, building and maintaining relationships with community partners is *too* time consuming in implementation research. Implementation researchers are often faced with the challenge of conducting rigorous data collection, analysis, and dissemination of findings in short time frames. Further, with the emphasis on speeding up adoption of EBIs, rapid research techniques are increasingly being used in health services, and implementation, research [48]. Collaboration, co-production, and user-led research may not be compatible with these rapid research techniques. Future research is therefore needed to examine and evaluate whether it is possible to incorporate meaningful PPIE in implementation research that utilises rapid research techniques. Nonetheless, given the reported benefits, we would recommend costing and factoring in researcher time to lay the groundwork and build partnerships, as this appears to be crucial to successful collaborations with public contributors in implementation research.

As discussed, most barriers were related to researchers and their attempts to build, maintain, and negotiate relationships with partners, including public contributors, over time. Financial remuneration, providing multiple opportunities for involvement, active listening and respecting the needs of partners, meeting contributors where they are, and regular contact with public contributors overcame these challenges. These barriers and enablers are consistent with reviews on PPI in health and social care research [9, 26], and knowledge translation research [49]. Notably however, reporting of barriers and facilitators varied across the included studies.

Studies were also inconsistent in describing and reporting their PPIE activities. Several studies did not provide details of who were involved or how often; and three included papers were protocols and therefore only outlined planned PPIE. Furthermore, while evaluation was not a focus of this scoping review, very few studies evaluated their use of PPIE, despite several studies highlighting the importance of evaluation and continuous monitoring [35, 40, 41, 43, 44]. Poor reporting, or the absence of reporting entirely, may account for why only 12 studies were included, as it was challenging to identify eligible studies. Moreover, we would argue if PPIE in implementation research is not reported in peer-reviewed articles, developments in this area will not be made. Future studies describing and evaluating the execution of PPIE in implementation research are therefore recommended. Surveys for researchers and contributors could be used to evaluate the use of PPIE [50], including the impact on research and the implementation of EBIs, and the impact on community members.

Papers were mostly excluded as they were not reporting on implementation research (*n* = 96). However, a large proportion of papers (*n* = 40) were excluded as they exclusively involved partners other than patients or members of the public; mostly healthcare professionals. All of the 12 included studies involved multiple collaborators, including healthcare professionals. This suggests power imbalances persist in implementation science, whereby healthcare professionals are still considered deliverers of EBIs, and are the core audience of implementation research. Further, because the included studies involved healthcare professionals as well as public contributors, which were often collectively referred to as “stakeholders” or “partners” in publications, it was occasionally difficult to extract findings for this review; particularly when attempting to make distinctions regarding the impact of patient and public involvement on the research and implementation of the EBIs.

This review is also limited by the relatively small number of included studies. As a result, it is not possible to make generalisable inferences regarding differences between geographical locations and settings, other than the observation that vocabulary across countries is inconsistent. This includes how levels of involvement differed by settings, and by PPIE activities. Notwithstanding this limitation, the review offers valuable insights into the execution of PPIE in implementation research, and highlights gaps in the knowledge base. Furthermore, the strengths of this review includes its comprehensive literature search strategy and use of two reviewers – both experienced in qualitative analysis and implementation science – to independently extract and analyse the data. This ensured the extraction and synthesis of the data were appropriate.

## Conclusion

This scoping review provides a systematic overview of the use of PPIE in the design and conduct of implementation research promoting the adoption of EBIs into healthcare. We also aimed to identify the factors that enable or hinder the involvement of patients and the public in the implementation of EBIs, and the reported benefits to the research, researchers, and public contributors. Our findings show many different types of partners were engaged in implementation research, including healthcare professionals alongside lay members. Our findings also show PPIE in implementation research is resource and time intensive. However, engaging the public in the design and implementation of community-based programs and trials may enhance cultural appropriateness, and therefore the sustainability of innovations. Key steps to improve PPIE in implementation research includes offering financial remuneration, providing multiple opportunities for involvement, active listening and respecting the needs of partners, and regular contact with public contributors. Furthermore, our understanding of the impact of PPIE in implementation research could be improved by better reporting and evaluation of activities in peer-reviewed articles.

## Supplementary Information

Additional file 1.

## Data Availability

The datasets used and/or analysed during the current study are available from the corresponding author on reasonable request.

## Abbreviations

CAB: Community Advisory Board
CBO(s): Community-based Organisation(s)
CBPR: Community-based Participatory Research
Co-PI: Co-Principal Investigator
CRP(s): Carer Reference Panel(s)
EBI(s): Evidence-based Intervention(s)
NAC: National Advisory Committee
PCOR: Patient-centred outcome research
PPI: Patient and Public Involvement
PPIE: Patent and Public Involvement and Engagement
UCD: User-centred design
UK: United Kingdom
USA: United States of America
